# Suppression of autophagy enhances preferential toxicity of paclitaxel to folliculin-deficient renal cancer cells

**DOI:** 10.1186/1756-9966-32-99

**Published:** 2013-12-04

**Authors:** Qi Zhang, Shuhui Si, Sue Schoen, Jindong Chen, Xun-Bo Jin, Guan Wu

**Affiliations:** 1 Minimally Invasive Urology Center, Provincial Hospital Affiliated to Shandong University, Jinan 250021, China; 2 Department of Urology, University of Rochester Medical Center, 601 Elmwood Avenue, Box 656, 14642 Rochester, NY, USA; 3 Pathology, University of Rochester Medical Center, Rochester, NY, USA; 4 Wilmot Cancer Center, University of Rochester Medical Center, Rochester, NY, USA

**Keywords:** Autophagy, Apoptosis, Paclitaxel, Taxol, Folliculin, FLCN, BHD, Kidney cancer

## Abstract

**Background:**

Paclitaxel, a widely used chemotherapeutic drug, can induce apoptosis in variety of cancer cells. A previous study has shown preferential toxicity of paclitaxel to FLCN-deficient kidney cancer cell line, UOK257. In this report, we investigate the cellular and molecular mechanism of paclitaxel-induced autophagy and apoptosis in renal cancer cells with and without FLCN expression.

**Methods:**

Two pairs of cell lines were used: FLCN siRNA-silenced ACHN cell line (ACHN-5968) and scrambled ACHN cell line (ACHN-sc); FLCN-null UOK257 cell line and UOK257-2 cell line restored with ectopic expression of FLCN. Autophagy was examined by western blot, GFP-LC3, transmission electron microscopy, and MDC assay. Cell viability and apoptosis were detected using MTT assay, DAPI stain and TUNEL assay. After inhibition of autophagy with 3-Methyladenine (3-MA) or Beclin 1 siRNA, cell viability and apoptosis were measured by MTT assay and TUNEL assay.

**Results:**

After paclitaxel treatment, a dose-dependent decrease in cell viability and increase in apoptosis were observed in FLCN-deficient UOK257 and ACHN-5968 cells compared to their FLCN-expressing counterparts, suggesting that renal cancer cells without FLCN were more sensitive to paclitaxel. Enhanced autophagy was found to be associated with paclitaxel treatment in FLCN-deficient RCC cells. The MAPK pathway was also identified as a key pathway for the activation of autophagy in these kidney cancer cells. Inhibition of phosphorylated ERK with ERK inhibitor U0126 showed a significant decrease in autophagy. Furthermore, after inhibition of autophagy with 3-Methyladenine (3-MA) or Beclin 1 siRNA, apoptosis induced by paclitaxel was significantly increased in FLCN-deficient UOK257 and ACHN-5968 cells.

**Conclusions:**

Preferential toxicity of paclitaxel to FLCN-deficient kidney cancer cells is associated with enhanced autophagy. Suppression of autophagy further enhances paclitaxel-induced apoptosis in FLCN-deficient renal cancer cells. Our results suggest that paclitaxel combined with an autophagy inhibitor might be a potentially more effective chemotherapeutic approach for FLCN-deficient renal cancer.

## Introduction

The functional connection between apoptosis and autophagy is a burgeoning area of research and has drawn intense interest from cancer researchers [1-3]. While apoptosis involves the activation of catabolic enzymes in signaling cascades that lead to destruction of cellular structures and organelles resulting in cell death, autophagy involves the formation of autophagosomal vesicles that engulf unwanted cellular components and impaired organelles and fuse with lysosomes for degradation and recycling [2]. Autophagy has been demonstrated to be involved in a wide variety of cellular processes, including cellular homeostasis, energy metabolism, cell death, cell survival, tissue regeneration, etc. Not surprisingly, autophagy plays critical roles in human disease processes, including cancer, neurodegenerative diseases, metabolic disorders, aging, infection and immunity [2].

It appears that the same stimuli, such as anticancer agents, can induce both apoptosis and autophagy in cells [1]. The role of autophagy in cancer cells is complex. As a nonapoptotic form of programmed cell death, the induction of autophagy in cancer cells may lead to cell death and therefore have a therapeutic effect on cancer cells [3]. However, autophagy could be activated under stress such as nutrient deprivation and hypoxia, playing an important role in cellular protection and cell survival [4]. Studies have shown that such cellular protection and survival endowed by autophagy might make cancer cells resistant to chemotherapy [5]. Therefore, it is essential to determine the function of autophagy in the process of anticancer therapy and its connection with apoptosis. Recently, although several pathways that link between the apoptotic and autophagic machineries have been reported, a strong causal relationship or interplay between autophagy and apoptosis in cancer cells has not been demonstrated adequately [1,2]. The therapeutic potential of induction or suppression of autophagy in cancer treatment undoubtably depends on understanding the role of autophagy in cancer cells.

Paclitaxel (Taxol) is an effective mitotic inhibitor and apoptosis inducer. It has been widely used in chemotherapy for lung cancer, breast cancer, ovarian cancer, and Kaposi’s sarcoma [6]. It has been shown that in non-small cell lung carcinoma cells, while paclitaxel treatment leads to apoptosis, paclitaxel also induces an autophagic response that plays a protective role impeding the eventual cell death [7]. While some recent studies demonstrated that paclitaxel treatment led to increased autophagy in lung cancer cells and osteosarcoma cells, and inhibition of autophagy increased the cytotoxic sensitivity of cells to paclitaxel [7,8], Veldhoen et al. reported that paclitaxel could inhibit autophagy in breast cancer cells by blocking activation of the class III phosphatidyl inositol 3 kinase, Vps34, and autophagy sensitized cells to paclitaxel toxicity [9]. These conflicting results suggested that the treatment effects of paclitaxel on autophagy might be cell-type dependent. Recently, it has been demonstrated that paclitaxel exhibits preferential toxicity to folliculin (FLCN)-deficient renal cell carcinoma (RCC) line, UOK257, a cell line which originated from a patient with Birt–Hogg–Dube (BHD) syndrome [10]. BHD syndrome, caused by *FLCN* mutations, is an autosomal dominant genetic disease characterized by susceptibility to renal cancer, renal and pulmonary cysts, and noncancerous tumors of the hair follicles [11]. Function of FLCN has been linked to mTOR and AMPK signaling pathways [12,13]. In addition, FLCN was reported to be involved in apoptosis [12,14-16]. Furthermore, FLCN was recently found to be associated with the activity of LC3-mediated autophagic program [17]. These findings might provide new insights into the treatment of BHD disease. While early-stage bilateral renal cancer associated with BHD disease could be managed with partial nephrectomy, an effective cure for BHD disease associated renal cancer has not been established. The preferential toxicity of paclitaxel to UOK257 FLCN-deficient cell line suggested that paclitaxel might be a candidate anticancer drug for FLCN-deficient tumors [10]. To further determine the cellular response of FLCN-deficient cell lines treated with paclitaxel, here we examined apoptosis and autophagy induced by paclitaxel in human renal cancer cell lines with or without FLCN expression. Our results indicated that autophagy induced by paclitaxel in FLCN-null renal cancer cells plays a protective role, and the inhibition of autophagy could increase apoptosis induced by paclitaxel treatment in these cancer cells.

## Materials and methods

### Reagents and antibodies

Dulbecco’s modified Eagle’s medium (DMEM) and fetal bovine serum (FBS) were purchased from Gibco (GIBCO, NY, USA). 3-Methyladenine (3-MA) was purchased from Sigma (Sigma-Aldrich, USA) and prepared as a stock solution of 100 mM in phosphate buffered saline (PBS). Paclitaxel, monodansyl cadaverine (MDC), and bafilomycin A1 were purchased from Sigma. U0126 was purchased from LC laboratories (LC Labs, USA). GFP-LC3 plasmid was obtained from Addgene (Addgene plasmid 24920). HT TiterTACSTM Assay Kit was purchased from TREVIGEN (TREVIGEN, USA), Beclin 1 siRNA was purchased from Invitrogen (Invitrogen Life Technologies, NY, USA). Antibodies used in this study included the following: Anti-cleaved Caspase-3, anti-MEK1/2, anti-phospho-MEK1/2, anti-phospho-ERK1/2, anti-p62 and anti-Beclin 1 (Cell Signaling Technology, USA); anti- LC3 polyclonal (Thermo Fisher Scientific, USA); anti-FLCN antibody (Obtained from the Van Andel Research Institute).

### Cell culture

Two pairs of cell lines were used: FLCN siRNA-silenced ACHN-5968 cell line and scrambled ACHN line (ACHN-sc); FLCN-null UOK257 cell line and UOK257-2 line restored with ectopic expression of FLCN. ACHN was purchased from ATCC, and ACHN-5968 was generated in our lab. UOK257 cell line was obtained from NCI, and UOK257-2 was prepared in our lab. All of these cell lines were cultured in DMEM medium, supplemented with 10% fetal bovine serum (FBS) and maintained at 37°C with 5% CO_2._

### Cell viability assay

The viability of cells was measured by MTT assay. Approximately 2 × 10^3^ cells were cultured in 96-well plates and treated with various reagents. MTT (5 mg/ml) was added to each well and cells were cultured at 37°C for 4 hours. Supernatant was removed and 200 μl DMSO per well was added to dissolve the formazan. Absorbance was measured at 570 nm using a microplate reader (BioTek).

### Western blot

Cells were harvested and lysed on ice for 45 min in RIPA lysis buffer (1 M Tris, PH7.4, 50 mM; NaCl 150 mM; 1%NP-40; EDTA 1 mM, plus standard protease inhibitor). The concentration of protein was measured by Nanodrop (Thermo). Equal amounts of total protein extracts were loaded and separated in 10% -15% SDS-PAGE gel and transferred to PVDF membranes. The membranes were blocked in Tris-buffered saline-Tween-20 (TBST) with 5% milk for 1 hour and incubated overnight at 4°C with different primary antibodies: mouse monoclonal anti-FLCN at a dilution of 1:1000, rabbit polyclonal anti-LC3-I/II (1:2000), rabbit polyclonal anti-p62 (1:2000), rabbit monoclonal anti-cleaved caspase-3 antibody (1:1500); mouse polyclonal anti-MEK (1:2000), rabbit polyclonal anti-phospho-MEK (1:2000); rabbit polyclonal anti-phospho-ERK (1:2000) or mouse monoclonal anti-Beclin 1(1:2000). The membranes were washed in TBST and incubated with secondary antibody at room temperature for two hours. Proteins were detected with ChemiDoc detection system (Bio-Rad).

### DAPI stain and TUNEL assay

Cell apoptosis was detected using DAPI stain and TUNEL assay. Cells with indicated reagents treatment were fixed with methanol/acetone (1:1) for 5 min at room temperature, then washed with phosphate-buffered saline (PBS) and stained with DAPI (1:2000 dilution, in 1× PBS) for 10 min. The cells were subsequently rinsed with PBS and observed under a fluorescent microscope (ZEISS).

To do the TUNEL assay , monolayer cells in 96-well plate were treated with corresponding reagents and cultured at 37°C. Cells were subsequently fixed in 3.7% paraformaldehyde for 7 minutes, and quantitation of apoptotic cells was measured by in situ colorimetric TUNEL assay (HT TiterTACSTMAssay kit, TREVIGEN®) following the manufacturer’s protocol. The results were immediately analyzed at 450 nm in the microplate reader.

### Autophagy assay

Autophagy was detected by transmission electron microscopy, GFP-LC3 and MDC assays. For transmission electron microscopy assay, cells were trypsinized, fixed for 24 hours with 2.5% glutaraldehyde in 0.1 M sodium cacodylate, and then fixed for another 30 minutes with 1.0% osmium tetroxide. Cells were trapped in agarose, treated with 0.5% uranyl acetate for 1 hour in the dark and dehydrated in a graded series of ethanol. They were transitioned to propylene oxide, infiltrated in Epon®/Araldite® resin for 24 hours, embedded in molds and polymerized for 48 hours at 70°C. Blocks were cut to determine area into 70 nm sections. The thin sections were collected on mesh nickel grids and stained with aqueous uranyl acetate and lead citrate. Grids were examined and photographed with a H-800 transmission electron microscope (Hitachi, Tokyo, Japan).

For GFP-LC3 assay, cells were cultured in 6-well plates and transfected with GFP-LC3 (Addgene plasmid 24920**)** with Lipofectamine™ 2000 (Invitrogen, USA) following the manufacturer’s protocol. At 24 hours after transfection, the cells were treated with paclitaxel (100 nM) or DMSO control and cultured at 37°C for 24 hours. The cells were subsequently examined under the fluorescence microscope (ZEISS), with 395 nm excitation wavelength and 509 nm emission filter respectively.

For MDC assay, cells cultured in 6-well plate were treated with 0.05 mM MDC and incubated at 37°Cfor 20 minutes. After staining, cells were fixed in 4% paraformaldehyde for 10 minutes and intracellular autophagy was detected using a fluorescence microscope (ZEISS) with 380 nm excitation wavelength and 525 nm emission filter.

MDC and GFP-LC3 assay results were ranked by the intracellular punctuates per cell: 1—0 to 4 punctuates, 2—5 to 9, 3—10 to 14, 4—15 to 19 and 5—more than 19. Cell scores were non-normally distributed and shown as mean of at least 20 per group, and confirmed by at least three separate experiments [18].

### Beclin 1 siRNA transfection

Cells were seeded in 6-well plates and incubated for 24 hours, then transfected with beclin 1-targeted siRNA or control random siRNA(Invitrogen) using Lipofectamine™ 2000 according to the manufacturer’s protocol. At 24 hours after transfection, cells were treated with or without paclitaxel for additional 24 hours and collected for western blot. Transfected cells were also used for MTT and TUNEL assays.

### Statistical analysis

Statistical significances were analyzed by ANOVA and paired Student *t* test with Statistics Package for Social Science (SPSS) software (Version 14). Qualitative data were expressed as means ± S.D, and p < 0.05 was considered statistically significant difference.

## Results

### Paclitaxel induced cytotoxicity and apoptosis in FLCN-deficient renal cancer cells

**Figure 1:**
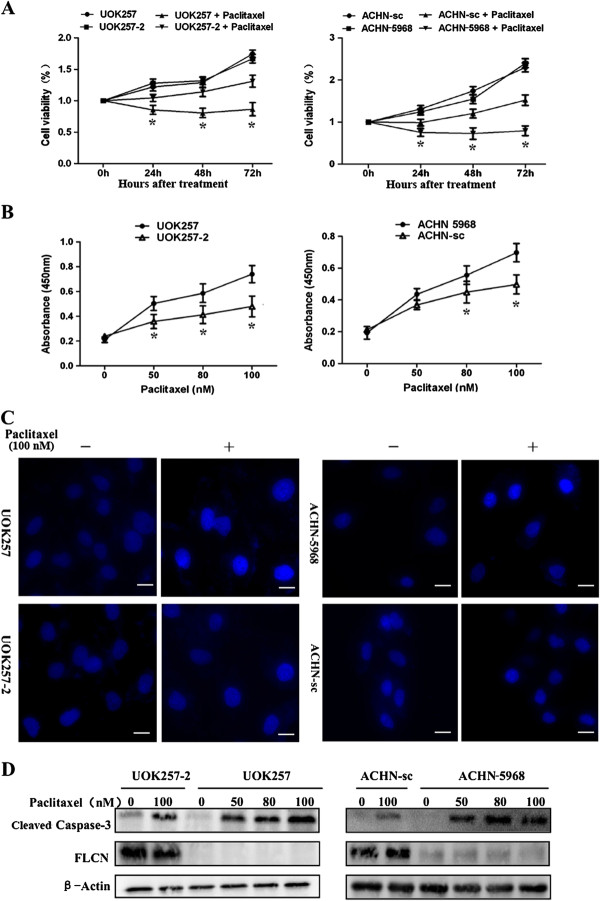
**Paclitaxel induced cytotoxicity and apoptosis in FLCN-deficient renal cancer cells. A**. Cells were treated with 100 nM paclitaxel or a control vehicle, cell viability was measured by MTT assay. Compared with UOK257-2 and ACHN-sc cells, FLCN-deficient UOK257 and ACHN-5968 cells were more sensitive to paclitaxel-mediated cytotoxicity. (*: p < 0.05. UOK257 with Paclitaxel vs UOK257-2 with Paclitaxel; ACHN-sc with Paclitaxel vs ACHN 5968 with Paclitaxel; n = 15) **B**. Cells were treated with 50, 80, and 100 nM paclitaxel for 24 hours. TUNEL assay was used for apoptosis analysis. FLCN-deficient cells (UOK257 and AHN-5968) showed more cell death compared to FLCN-expressing counterparts. (*: p < 0.05. UOK257 vs UOK257-2; ACHN-sc vs ACHN 5968; n = 15). **C**. Cells treated with 100 nM Paclitaxel or a control vehicle were stained with DAPI and analyzed under fluorescence microscope. Bright blue fluorescent signals showed the damaged nuclear DNA due to apoptosis. More bright blue fluorescent spots were observed in FLCN-deficient cells. Scale bar = 10 μm. **D**. Cells were treated with 50, 80, and 100 nM paclitaxel for 24 hours, cleaved caspase-3 and FLCN protein were detected by western blot. Elevated cleaved caspase-3 expression was detected in FLCN-deficient cells.

To determine whether paclitaxel treatment leads to apoptosis in FLCN-deficient renal cancer cells, cell lines with (ACHN-sc and UOK257-2) and without (ACHN-5968 and UOK257) FLCN expression were treated with paclitaxel. The cell viability was analyzed by MTT assay after treatment. As shown in Figure 1A, suppression of cell growth by paclitaxel on FLCN-deficient UOK257 and ACHN-5968 cells was more significant than that on matched UOK257-2 and ACHN-sc cells, indicating more severe paclitaxel-induced cytotoxicity to FLCN-deficient cells. We further analyzed apoptosis in these cell line pairs by using in situ colorimetric TUNEL assay. As shown in Figure 1B, paclitaxel could induce apoptosis in all treated cells with or without FLCN expression. However, a much greater number of apoptotic cells were detected in UOK257 and ACHN 5968 lines compared to UOK257-2 and ACHN-sc lines. The differences were also dose-dependent and reached maximum at 100 nM of paclitaxel. After paclitaxel treatment, cell nuclear morphological changes were observed using DAPI staining assay (Figure 1C). Paclitaxel caused more apoptosis with destroyed DNA in UOK257 and ACHN 5968 cells (indicated as the strong blue fluorescence). Furthermore, after the treatment of paclitaxel, the 35 kDa protein caspase-3 was cleaved into 17 kDa fragments in cells with or without FLCN expression (Figure 1D). The levels of cleaved caspase-3 were obviously higher in UOK257 and ACHN 5968 cells upon the treatment with 100 nM paclitaxel, indicating more apoptosis was induced in cells without FLCN expression. These results supported the conclusion that paclitaxel induces more apoptosis in FLCN-deficient renal cancer cells.

### Paclitaxel induced autophagy in FLCN-deficient renal cancer cells

**Figure 2:**
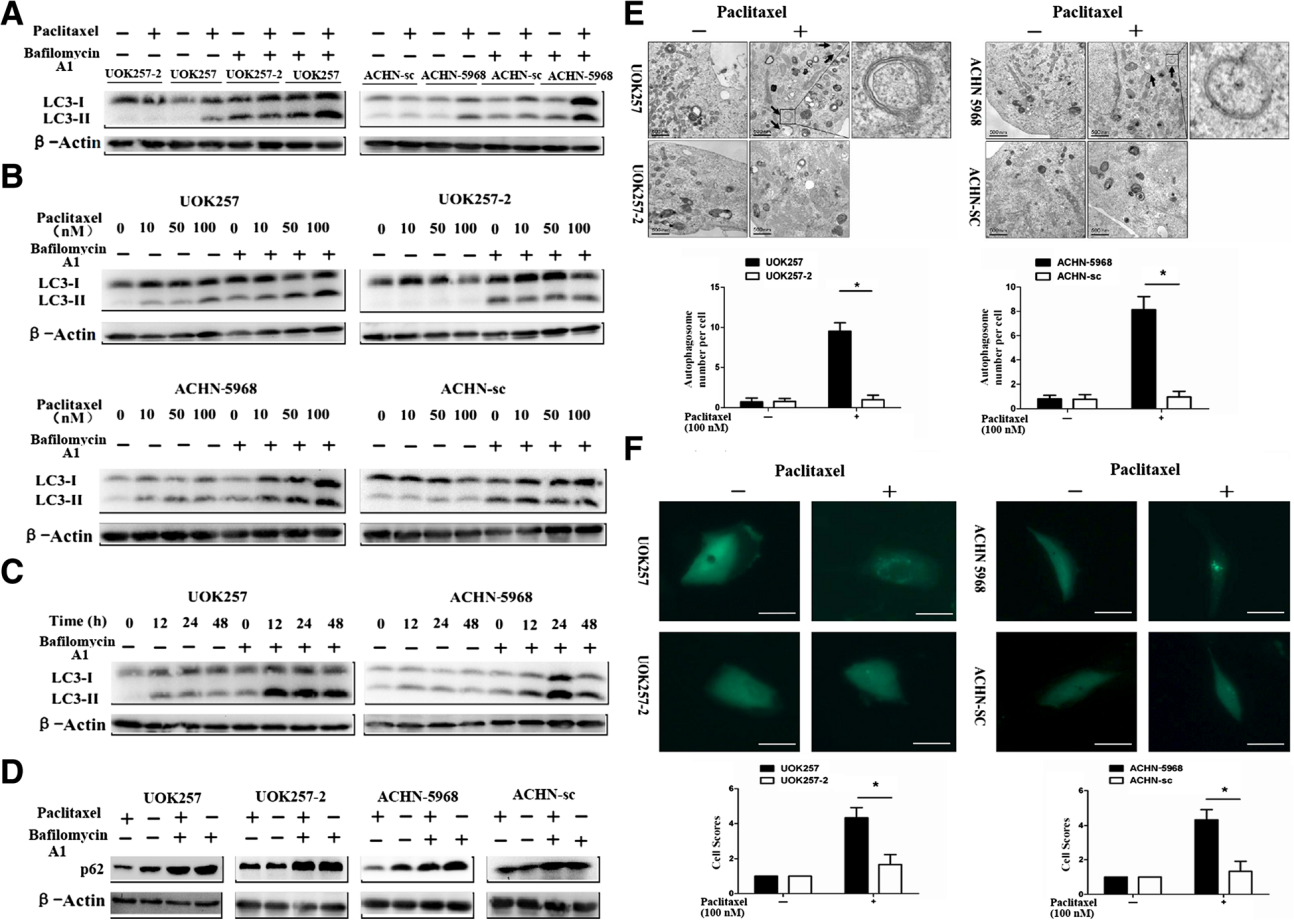
**Paclitaxel induced autophagy in UOK257 and ACHN-5968 cells. A**. UOK257/UOK257-2 and ACHN-sc/ACHN 5968 cells were treated with 100 nM paclitaxel for 24 hours. In the absence or presence 100 nM bafilomycin A1 for four hours before harvest, elevated LC3-II levels were detected in FLCN-deficient UOK257 and ACHN-5968 cells by Western blot assay. **B**. In the absence or presence bafilomycin A1, LC3 protein levels were examined for cells treated with paclitaxel at various concentrations for 24 h, the highest LC3 level was observed at 100 nM in FLCN-deficient cells. **C**. FLCN-deficient cells were treated with 100 nM paclitaxel and harvested at different time intervals with or without bafilomycin A1 treatment. LC3-II expression peaked at 24 h treatment. **D**. Cells were treated with 100 nM paclitaxel and harvested with or without bafilomycin A1 treatment. In the absence of lysosomal inhibitor bafilomycin A1, decreased p62 was observed in paclitaxel-treated FLCN-deficient cells. **E**. Paclitaxel-induced autophagosomes in cells were observed using transmission electron microscopy. Autophagosome formation was found in FLCN-deficient UOK257 and ACHN-5968 cells. Arrows indicate autophagosome structures. Scale bars = 500 nm (*: p < 0.05. UOK257 vs UOK257-2; ACHN-sc vs ACHN 5968; n = 30). **F**. Cells were transfected with GFP-LC3 and analyzed under fluorescent microscopy for autophagosomes (*: p < 0.05, UOK257 vs UOK257-2; ACHN-sc vs ACHN 5968; n = 60). Scale bars = 15 μm.

To determine whether paclitaxel induces autophagy as well in FLCN-deficient renal cancer cells, we measured the expression of microtubule-associated protein 1 light chain 3 (LC3) in paclitaxel-treated cells by Western blot. LC3 is an important autophagy marker recruited to the autophagosome membrane. LC3 has two isoforms, LC3-I and LC3-II. During autophagy, LC3-I is conjugated to autophagic membrane-associated phosphatidylethanolamine and converted to LC3-II. Increased LC3-II level, especially increased LC3-II/LC3-I ratio, may indicate the occurrence of autophagy [19,20]. To exclude the possibility that the increased LC3-II levels were resulted from the accumulation of LC3-II due to downstream inhibition other than paclitaxel induction, we treated the cells with paclitaxel in presence or absence of lysosomal inhibitor bafilomycin A1. As shown in Figure 2, although increased LC3-II levels were detected in all of the bafilomycin A1-treated cells due to inhibition of lysosomal degradation of LC3-II, LC3-II levels were even higher in the paclitaxel-treated FLCN-deficient cells compared to that in the FLCN-expressing cells regardless of balfilomycin A1 (Figure 2A). The paclitaxel-mediated LC3 expression levels were also measured at various drug concentrations and different time points with or without bafilomycin A1 treatment (Figure 2B, C). The paclitaxel treatment led to increase of LC3-II level in a dose-dependent manner and seemed to peak at 24 hours in FLCN-deficient cells. To further confirm that paclitaxel could induce autophagy in FLCN-deficient cells, we examined the p62 expression by Western blot. The reduced p62 level usually indicates activation of autophagy in cells [19,21]. In the absence of lysosomal inhibitor bafilomycin A1, we observed that expression of p62 protein was decreased in paclitaxel-treated FLCN-deficient cells, suggesting that autophagy was activated and the p62 protein was degraded via autophagy (Figure 2D). The p62 level was obviously elevated in FLCN-deficient cells treated with bafilomycin A1 and paclitaxel, indicating autophagy was blocked by bafilomycin A1 and p62 was accumulated in these cells (Figure 2D) These results demonstrated that paclitaxel could induce autophagy in FLCN-deficient cells.

To further confirm the induction of autophagy in these cells, we examined the autophagosome formation after paclitaxel treatment using three assays. First, we examined the autophagosome formation with transmission electron microscopy assay. Both pairs of cell lines were examined after paclitaxel treatment. The results showed that increased autophagosome numbers were present in FLCN-deficient cells (UOK257 and ACHN-5968) (Figure 2E). We next examined the formation of autophagosome through the appearance of the punctate structures with GFP-LC3 assay. We transfected these cells with a GFP-LC3 plasmid that ectopically expressed LC3 in the affected cells. The results showed that the FLCN-deficient cells exhibited a higher number of punctate structures compared to FLCN-expressing UOK257-2 and ACHN-sc cells (Figure 2F). We further detected autophagy in cells with monodansyl cadaverine (MDC) staining assay. Since MDC was demonstrated to have higher affinity for lysosomes, here we used it as an auxiliary means [22]. Similar to the GFP-LC3 assay, we analyzed the formation of autophagosomes under fluorescence microscopy. Again, the FLCN-deficient cells displayed much higher number of punctate structures compared to corresponding counterparts (Additional file 1: Figure S1). These results showed that autophagy was induced by paclitaxel treatment in FLCN-deficient cells.

### Paclitaxel induces autophagy in FLCN-deficient cells via activation of ERK pathway

**Figure 3:**
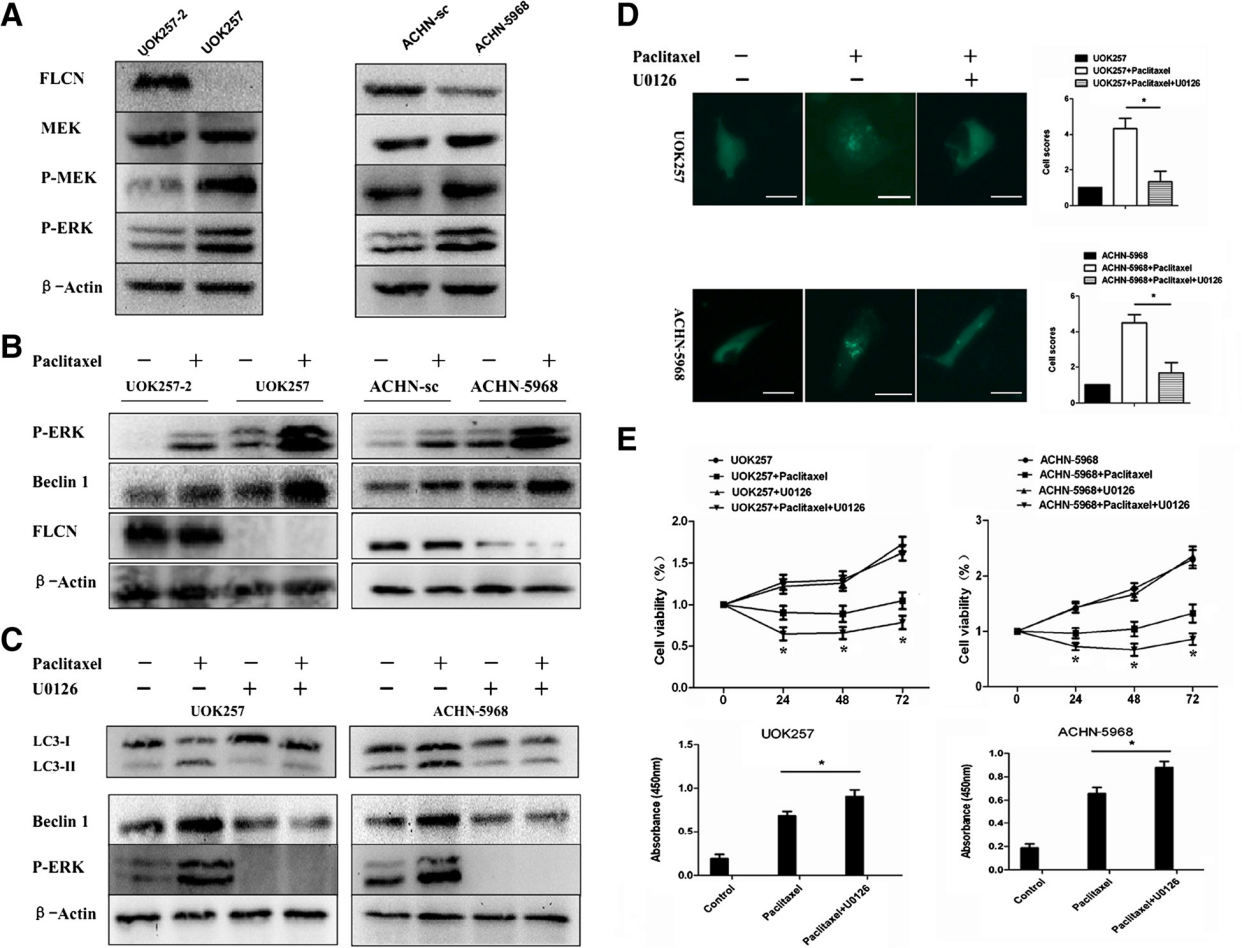
**FLCN reversely regulated paclitaxel-induced autophagy via the ERK 1/2 pathway. A**. ERK 1/2 pathway was activated in UOK257 and ACHN-5968 cells. Both P-MEK and P-ERK were increased those cells. **B**. Western Blot analysis showed that both P-ERK and Beclin 1 proteins were significantly elevated in FLCN-deficient cells after paclitaxel, compared to controls. **C**. ERK inhibitor U0126 repressed the expression of LC3-II protein in FLCN-deficient cells. **D**. Fewer punctuated dots were detected in GFP-LC3 transfected FLCN-deficient cells after treatment of paclitaxel and U0126 (*: p < 0.05, UOK257 + Paclitaxel vs UOK257 + Paclitaxel + U0126; ACHN 5968 + Paclitaxel vs ACHN 5968 + Paclitaxel + U0126; n = 60). Scale bars = 15 μm. **E.** Treatment with U0126 further enhanced preferential toxicity of paclitaxel to FLCN-deficient cells (*: p < 0.05. UOK257 + Paclitaxel vs UOK257 + Paclitaxel + U0126; ACHN 5968 + Paclitaxel vs ACHN 5968 + Paclitaxel + U0126; n = 15). After treatment with U0126, apoptosis induced by paclitaxel was significantly increased in FLCN-deficient UOK257 and ACHN-5968 cells (*: p < 0.05. UOK257: Paclitaxel vs Paclitaxel + U0126; ACHN 5968: Paclitaxel vs Paclitaxel + U0126; n = 15).

To explore the molecular mechanism of paclitaxel induced autophagy in FLCN-deficient cells, we examined the alteration of the ERK pathway, which is known to be associated with autophagic regulation in lung cancer cells [23,24]. As presented in Figure 3, elevated expressions of phospho-MEK and phospho-ERK were detected in FLCN-deficient renal cell lines, indicating that absence of FLCN was connected with the activation of the ERK pathway (Figure 3A). Paclitaxel treatment further significantly increased the expression of phospho-ERK and Beclin 1 in FLCN-deficient UOK257 and ACHN-5968 cells. Only slightly elevated phospho-ERK and Beclin 1 were observed in FLCN-expressing cells (Figure 3B). Additionally, treatment with the ERK inhibitor U0126 significantly reduced the expression of LC3, Beclin 1, and phospho-ERK in UOK257 and ACHN-5968 cells (Figure 3C, D). In addition, U0126 treatment further enhanced the cytotoxicity and apoptosis induced by paclitaxel in these FLCN-deficient cells (Figure 3E, F). These results further suggested that paclitaxel induced autophagy in FLCN-deficient cells via the ERK pathway.

### Inhibition of autophagy enhanced paclitaxel-induced apoptosis in FLCN-deficient cells

**Figure 4:**
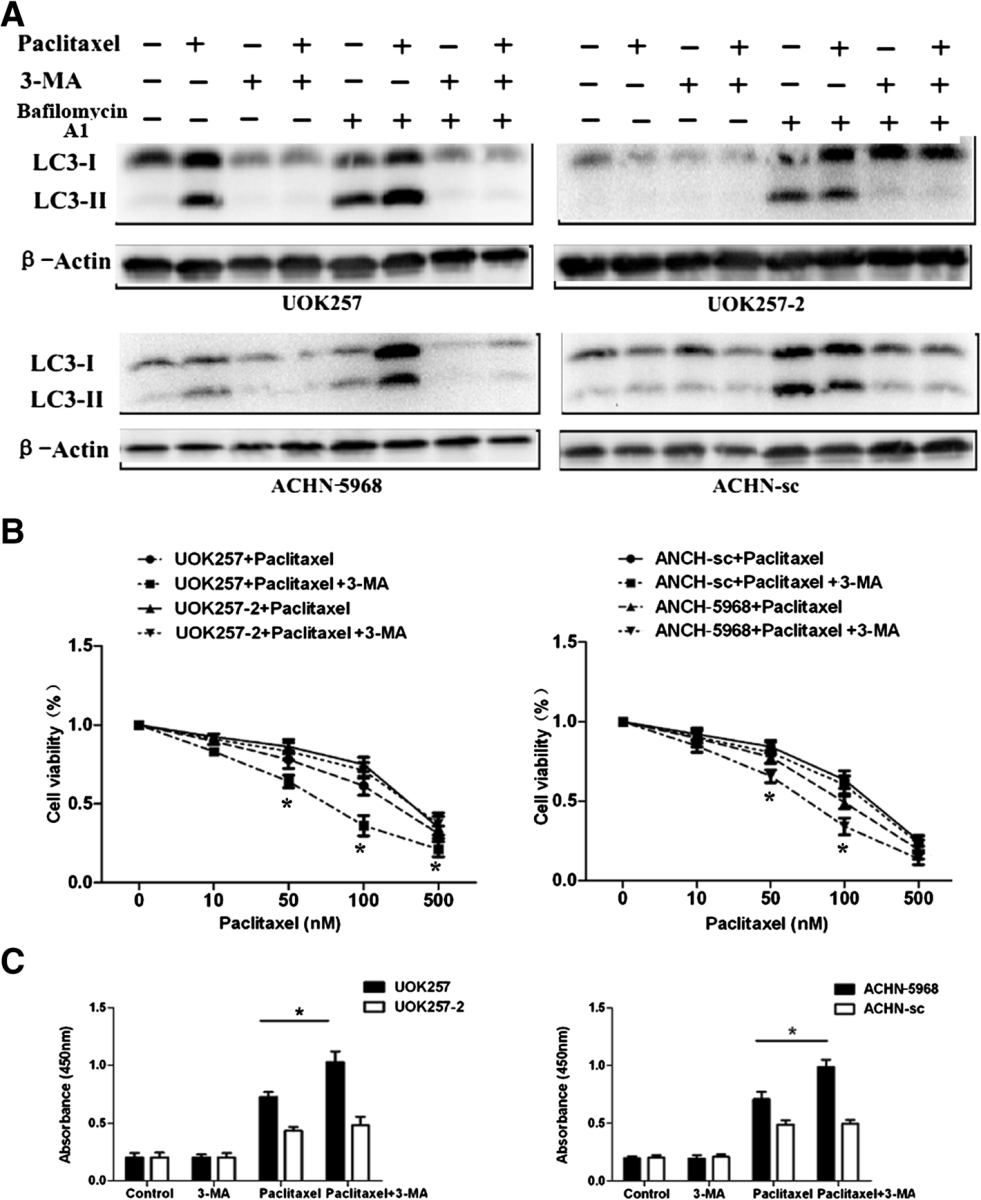
**3-MA inhibited autophagy and enhanced apoptosis induced by paclitaxel treatment in FLCN-deficient cells. A**. Cells were pretreated with 5 mM 3-MA for 3 hours and subsequently treated with 100 nM paclitaxel or a control vehicle for 24 hours with or without bafilomycin A1 treatment. LC3 proteins were dramatically decreased after autophagy inhibitor 3-MA. **B**. Cells were treated with 3-MA and different concentrations of paclitaxel, MTT assay showed that cell viability was more significantly reduced in FLCN-deficient cells compared to 3-MA untreated cells (*: p < 0.05. UOK257 + Paclitaxel vs UOK257 + Paclitaxel + 3-MA; ACHN 5968 + Paclitaxel vs ACHN 5968 + Paclitaxel + 3-MA; n = 15). **C.** TUNEL assay showed that more apoptotic cells were detected among FLCN-deficient cells treated with 3-MA and paclitaxel (*: p < 0.05. UOK257: Paclitaxel vs UOK257+ 3-MA; ACHN 5968: Paclitaxel vs Paclitaxel + 3-MA; n = 15).

To determine the impact of autophagy on paclitaxel-mediated FLCN-deficient cell death, we applied autophagy inhibitor 3-MA or Beclin 1 siRNA to suppress autophagy in those cell lines. As showed in Figure 4A, pretreatment with 5 mM 3-MA led to a significant decrease of LC3-II levels in FLCN-deficient UOK257 and ACHN-5968 cells, indicating that autophagy was inhibited by 3-MA in those cells. No obvious LC3-II changes were observed in FLCN-expressing cell lines (UOK257-2 and ACHN-sc) with 3-MA treatment. Pretreatment with 3-MA effectively inhibited cell viability and enhanced paclitaxel-mediated apoptosis in UOK257 and ACHN-5968 cells compared to UOK257-2 and ACHN-sc cells (Figure 4B, C). These results demonstrated that inhibition of autophagy could enhance paclitaxel-mediated apoptosis and cytotoxicity in FLCN-deficient renal cancer cells.

### Beclin 1 knockdown inhibited autophagy and sensitized FLCN-deficient cells to paclitaxel

**Figure 5:**
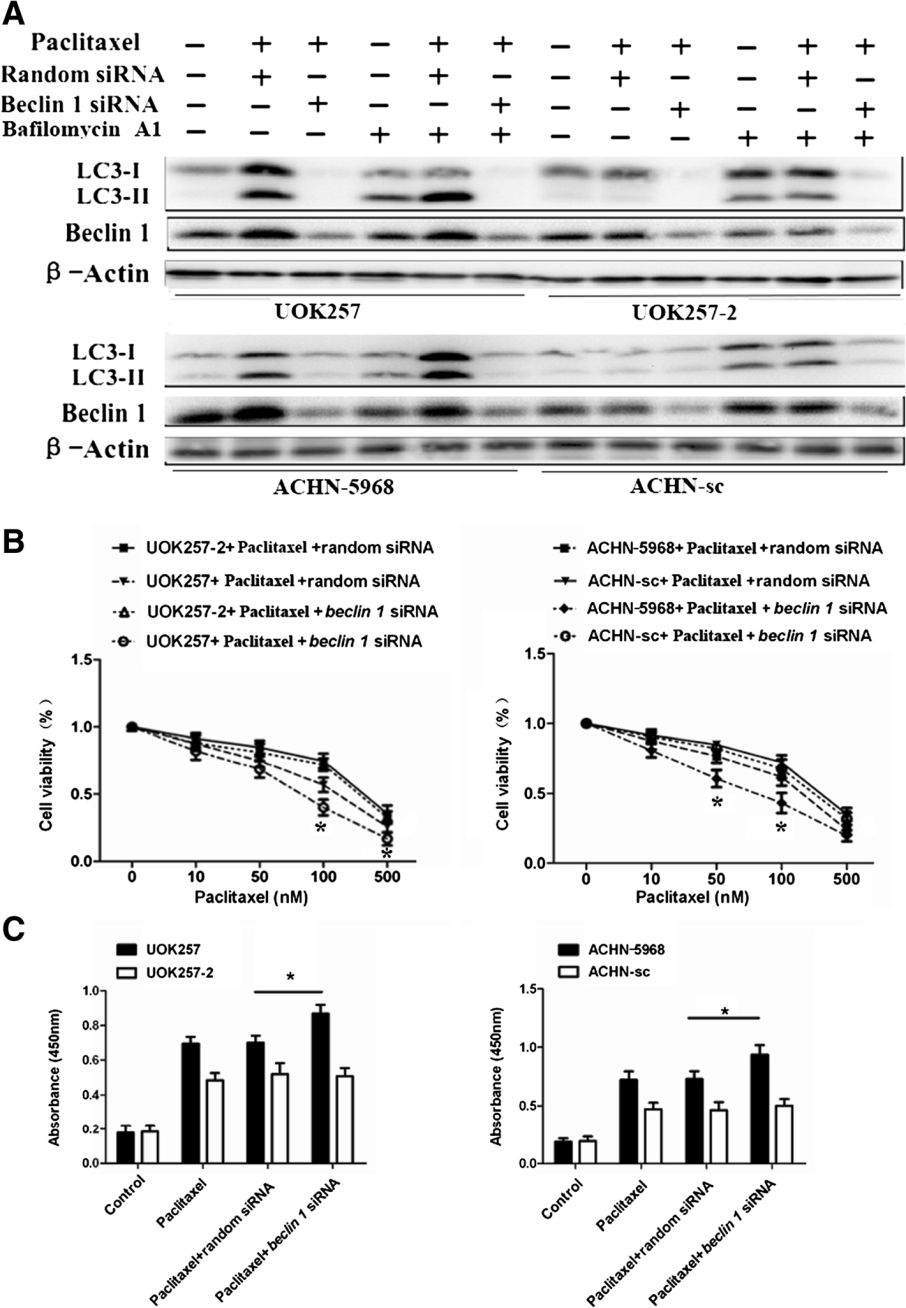
**Beclin 1 knockdown inhibited autophagy and sensitized FLCN-deficient cells to paclitaxel. A**. Cells were transfected with Beclin 1 siRNA or a random siRNA control for 24 hours and subsequently treated with 100 nM paclitaxel for 24 hours with or without bafilomycin A1 treatment, LC3 protein levels were detected using Western blot. Less LC3 proteins were detected in Beclin 1 siRNA treated cells. **B**. FLCN-deficient cells transfected with Beclin 1 siRNA or a random siRNA control were treated with different concentrations of paclitaxel. MTT assay showed that cell viability was obviously decreased after Beclin 1 siRNA treatment (*: p < 0.05. UOK257 + Paclitaxel + random siRNA vs UOK257 + Paclitaxel + *beclin 1* siRNA; ACHN 5968 + Paclitaxel + random siRNA vs ACHN 5968 + Paclitaxel + *beclin 1* siRNA; n = 15). **C**. Number of apoptotic cells increased after treatment with Beclin 1 siRNA and 100 nM paclitaxel (*: p < 0.05. UOK257: Paclitaxel + random siRNA vs Paclitaxel + *beclin 1* siRNA; ACHN 5968: Paclitaxel + random siRNA vs Paclitaxel + *beclin 1* siRNA; n = 15).

To further confirm the role of autophagy on cell death, we knocked down another autophagy marker, Beclin 1, in all four cell lines by the siRNA method. UOK257, UOK257-2, ACHN-sc, and ACHN-5968 cells were transfected with Beclin 1 siRNA or a negative control siRNA, respectively. We then examined the effects of Beclin 1 knockdown on paclitaxel-mediated apoptosis and cell viability in these cells. Compared to the treatment with negative control siRNA, Beclin 1 siRNA remarkably abrogated the paclitaxel-induced LC3-II expression in FLCN-deficient UOK257 and ACHN-5968 cells regardless of bafilomycin A1treatment (Figure 5A). The knockdown of Beclin 1 led to a significant increase of apoptosis and inhibition of cell viability in FLCN-deficient cells, which was consistent with the results obtained through 3-MA treatment (Figure 5B, Figure 5C). These data indicated that autophagy provided protection and survival advantage to FLCN-deficient cells against cell apoptosis and cell death induced by paclitaxel. Inhibition of autophagy could increase the paclitaxel-induced cytotoxicity to these cells and might improve the efficacy of paclitaxel against these cancer cells.

## Discussion

As a cancer chemotherapeutic drug, paclitaxel has been widely used in chemotherapy for lung cancer, breast cancer, ovarian cancer, and Kaposi’s sarcoma [6]. Kidney cancers are known to be resistant to conventional chemotherapy [25-27]. Gemcitabine in combination with doxorubicin has only shown some benefit in patients with certain types of kidney cancer [28]. A recent study has shown preferential toxicity of mithramycin and paclitaxel to FLCN-deficient kidney cancer cell line, UOK257 [10]. If proven, this provides a unique therapeutic opportunity to a group of tumors related to BHD disease. In this study, we chose paclitaxel for further study its effects on FLCN-deficient kidney cancer cells to find a more effective way to treat these cancer cells. Besides FLCN-deficient cell line UOK257, a cell line derived from a BHD patient’s kidney cancer [29], we also employed a RCC cell line, ACHN, with known FLCN expression and its FLCN expression could be effectively suppressed with siRNA. Although ACHN cell line was not derived from a BHD patient and we would not expect that silencing FCLN with siRNA in ACHN cell line would replicate a RCC cell line derived from a BHD patient, our study did show consistent results between UOK257 and ACHN cells in respect to paclitaxel treatment-induced apoptosis and autophagy in the presence or absence of FLCN. We first demonstrated that paclitaxel could lead to apoptosis as well as autophagy in FLCN-deficient cell lines UOK257 and ACHN-5968. After paclitaxel treatment, a dose-dependent decrease in cell viability and increase in apoptosis were observed in both FLCN-deficient UOK257 and ACHN-5968 cells, while their FLCN-expressing counterparts showed relatively less changes. These results suggested that FLCN-deficient RCC cells were more sensitive to paclitaxel exposure through apoptosis, indicating that FLCN may play a role against paclitaxel-induced apoptosis. We further detected that enhanced autophagy occurred along with apoptosis after paclitaxel treatment in FLCN-deficient RCC cells compared to FLCN-expressing counterparts, suggesting that paclitaxel treatment could also induce autophagy in FLCN-deficient RCC cell lines. Previous studies have suggested that FLCN was involved in apoptosis. While Reiman et al. identified that FLCN might up-regulate the expression of a number of apoptosis genes and activates apoptosis [14]. Baba *et al*. found that FLCN interacted with the Bcl 2 family to inhibit apoptosis in B cells in FLCN knockout mouse [16]. Interestingly, FLCN, like tumor suppressor VHL, seems to be associated with the activity of LC3-mediated autophagic program, which suggests that the existence of functional crosstalk between two major tumor suppressors in renal cancer, VHL and FLCN, converging on regulation of autophagy [17]. Behrends *et al*. also suggested that FNIP1, a partner protein of FLCN, is a part of an autophagy interaction network [30]. Based on these reports and our data, it seems that the presence of FLCN can prevent cells from apoptosis and autophagy following paclitaxel treatment.

Since existing reports have presented conflicting results on the effects of paclitaxel treatment on autophagy in different cell types [7-9], it seems plausible that the effects of paclitaxel on autophagy is cell-type-specific. In addition, some specific proteins or signal pathways may influence the regulation of paclitaxel on autophagy and lead to different autophagic effects. It was reported that paclitaxel could induce autophagy only in Cdx1-expressing colon cancer cells, but not in Cdx1-deficient colon cancer cells [31]. In our study, we observed that autophagy was obviously activated by paclitaxel via the MAPK pathway and beclin 1 protein in FLCN-deficient renal cancer cells, but not in FLCN-expressing cells. These results demonstrated that paclitaxel treatment could specifically sensitize FLCN-deficient renal cancer cells to paclitaxel toxicity and induce autophagy in these cells.

In our study, we also found that the MAPK pathway was activated after paclitaxel treatment in FLCN-deficient RCC cells and that autophagy was significantly decreased after treatment with ERK inhibitor U0126 in these cancer cells. These results indicated that MAPK pathway played a key role in the activation of autophagy in these kidney cancer cells and inhibition of MAPK pathway reduced autophagy in these cells. To further determine whether paclitaxel treatment induced autophagy represents synergistic antineoplastic effects on FCLN-deficient RCC cells or provides a protective mechanism against apoptosis, we used autophagy inhibitor and Beclin 1 siRNA to suppress autophagy. Our experiments demonstrated that increased apoptosis was detected by direct inhibition of autophagy with 3-Methyladenine (3-MA) or Beclin 1 siRNA after paclitaxel exposure in FLCN-deficient UOK257 and ACHN-5968 cells. These results suggested that in FLCN-deficient RCC cells paclitaxel treatment-induced autophagy provided a protective mechanism against apoptosis and other damage. Based on mounting evidence, it is conceivable that autophagy induced by different chemotherapeutic agents plays different roles or opposite roles in different types of cancer. Genetic, epigenetic, and metabolic backgrounds of specific types of cancer are likely the keys to determine the role of autophagy during chemotherapy. For FLCN-deficient RCC cells, suppression of autophagy enhances preferential toxicity of paclitaxel.

## Conclusions

In summary, our data demonstrated that in FLCN-deficient renal cancer cells, paclitaxel treatment induced apoptosis is associated with increased autophagy that plays a protective role against the treatment. Inhibition of autophagy significantly enhanced paclitaxel-induced apoptosis. Our findings suggest that paclitaxel treatment combined with inhibition of autophagy might be a potentially more effective chemotherapeutic approach for FLCN-deficient renal cancer and BHD-related kidney tumors.

## Competing interests

The authors declare that they have no competing interests.

## Authors’ contributions

QZ and SHS performed the experiments. QZ, XBJ and GW designed the study. QZ and JDC performed data analysis. JDC and SS supervised the study. QZ, JDC, and GW wrote the manuscript. All authors read and approved the final manuscript.

## Supplementary Material

Additional file 1: Figure S1 Paclitaxel-induced autophagosomes in cells with or without FLCN expression were detected using MDC assay. Punctuated areas in cells represent autophagosomes. Cell scores were calculated by the intracellular punctuates. Scale bars = 10 μm (*: p < 0.05. UOK257 vs UOK257-2; ACHN-sc vs ACHN 5968; n = 60). Click here for file
