# Supplementary figures and images for: Suppression of autophagy enhances preferential toxicity of paclitaxel to folliculin-deficient renal cancer cells
Source: J Exp Clin Cancer Res. 2013 Dec 4;32(1):99. doi: 10.1186/1756-9966-32-99 (PMC3879005; doi:10.1186/1756-9966-32-99)

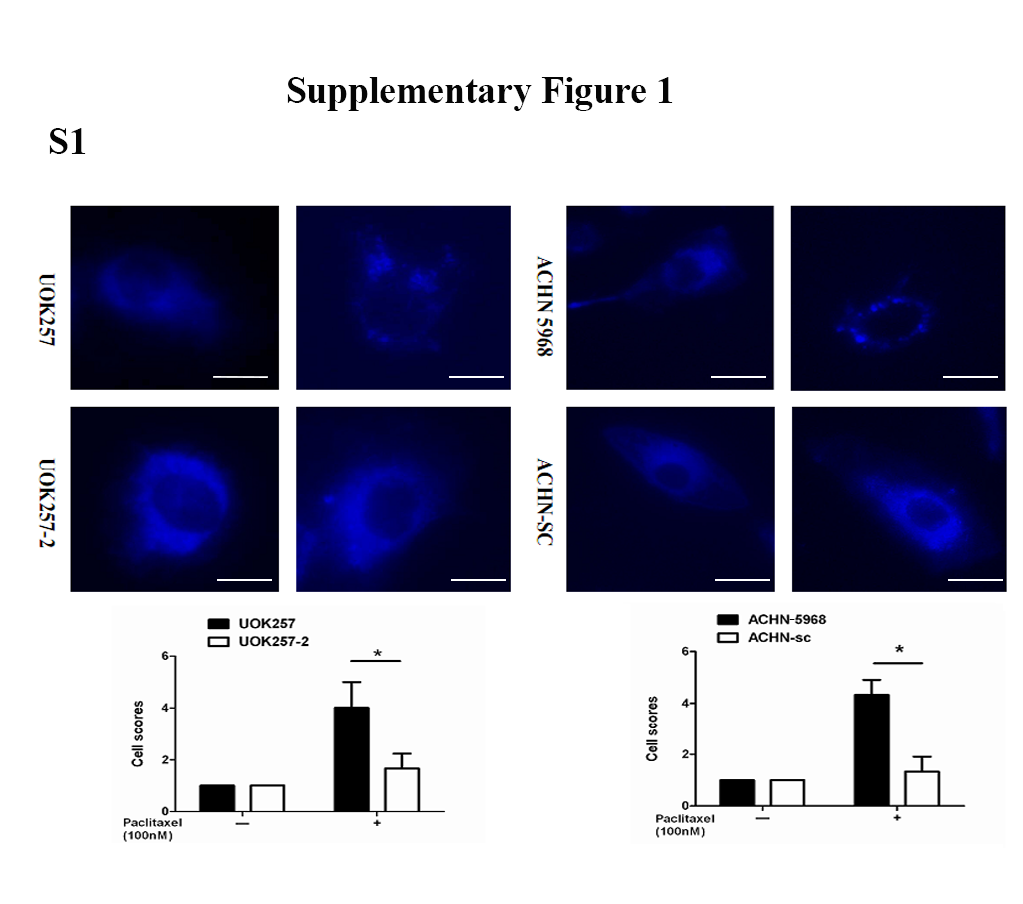

Supplement: Additional file 1: Figure S1 — Paclitaxel-induced autophagosomes in cells with or without FLCN expression were detected using MDC assay. Punctuated areas in cells represent autophagosomes. Cell scores were calculated by the intracellular punctuates. Scale bars = 10 μm (*: p < 0.05. UOK257 vs UOK257-2; ACHN-sc vs ACHN 5968; n = 60). [file 1756-9966-32-99-S1.tiff]
